# The Effect of Bioceramic HiFlow and EndoSequence Bioceramic Sealers on Increasing the Fracture Resistance of Endodontically Treated Teeth: An In Vitro Study

**DOI:** 10.7759/cureus.33051

**Published:** 2022-12-28

**Authors:** Mohamad Khir Abdulsamad Alskaf, Hassan Achour, Hasan Alzoubi

**Affiliations:** 1 Department of Endodontic and Operative Dentistry, Damascus University, Damascus, SYR; 2 Department of Pediatric Dentistry, Damascus University, Damascus, SYR

**Keywords:** mandibular premolars, ah plus, bioceramic hiflow, endosequence bioceramic, root fracture

## Abstract

Background/purpose

Root fractures after endodontic treatment are a serious complication, and it often causes tooth loss, several studies have found a correlation between root canal preparation and filling, and the possibility of root fracture. Therefore, this study aimed to evaluate the root fracture resistance of the endodontically treated mandibular premolars after preparing and filling by EndoSequence Bioceramic (BC), EndoSequence BC HiFlow, and AH Plus (AHP).

Materials and methods

The study sample consisted of 75 single-rooted and single-canal mandibular premolars, they were randomly distributed into five groups: Group I: root canals preparation and fillings with AHP sealer; Group II: root canals preparation and fillings with EndoSequence BC sealer; Group III: root canals preparation and fillings with BC HiFlow sealer; Group IV (Negative Control): without root canals preparation; and Group V (Positive Control): canals preparation without root canals filling. A glide path was established by #10 hand K-file, then the crowns were cut, and the canals were prepared and filled. All teeth were restored with glass ionomer cement and kept in an incubator at 37°C for a week. All samples were placed within acrylic bases and a vertical force was applied using a Testometric machine and the fracture resistance force was recorded.

Results

EndoSequence BC and BC HiFlow groups showed slightly better fracture resistance (494.440, 496.960 N respectively) than AHP group (492.680 N). However, no statistically significant difference was found between the two groups (P-value >0.05). The greatest mean fracture force was observed in the positive control group (736.040 N) with statistically significant difference between the other groups (P-value <0.01) and the least mean fracture force was shown in the negative control group (318.040 N) with statistically significant difference between the other groups (P-value <0.01).

Conclusion

Based on this in vitro study, the use of EndoSequence BC, BC HiFlow, and AHP enhanced the fracture resistance in root-filled single-rooted premolar teeth. While, the application of EndoSequence BC, BC HiFlow, and AHP did not increase the fracture resistance of roots compared to that of unprepared root canals.

## Introduction

Longitudinal fractures in endodontically treated teeth are a common complication that leads to teeth extraction [1]. The hardening of endodontically treated teeth depends on the amount of remaining dental tissue [2]. Therefore, the adhesion of filling pastes with dentin is an important issue in endodontic treatment, as it must form a single mass with dentin and filling materials in the canal space [3].

AH Plus (AHP) is a root canal filling paste consisting of bisphenol epoxy resin that is easily applied within the root canal and, when used in small quantities with gutta-percha, provides long-term stability [4]. Bioceramic (BC) is an injectable root canal filling material, containing zirconium oxide, calcium silicates, calcium phosphate monobasic, calcium hydroxide, filler, and thickening agents [5].

Endosequence BC Sealers have been introduced, which are characterized by biocompatibility, good sealing, and dimensional stability [5]. Endosequence BC Sealer pastes contain calcium phosphate, which leads to the formation of hydroxyapatite during setting and making bonds between dentin and filling materials, which increases the strength and durability of the tooth [6].

Recently Endosequence BC HiFlow pastes were also introduced, which have additional characteristics that allowed them to be used with various canal filling techniques such as warm filling, which were not usable with Endosequence BC Sealers, as its fluidity increases when exposed to heat, which increases the possibility of its inclusion within dentinal canals [7].

Therefore, this study was conducted to evaluate if there are no differences between these materials as filler pastes compared to AHP in increasing root fracture resistance.

## Materials and methods

This is an in vitro study to compare root fracture resistance between three filling pastes (AHP - Endosequence BC Sealers - Endosequence BC HiFlow) in extracted premolars for orthodontic reasons. The study protocol was approved by the Scientific Research and Postgraduate Board of Damascus University Ethics Committee of Damascus University, Damascus, Syria (IRB No. UDDS-568-22072019/SRC-3674). The sample size was determined using a sample size calculation program (PS Power and Sample Size Calculation Program, Version 3.0.43). The sample size was calculated using outcomes from Phukan et al., comparing the outcomes of four root canal filling pastes [8]. After calculating the sample size, the sample size required to detect a significant difference was 75 teeth (90% power, two-sided 5% significance level, and effect size 0.33).

The studied sample was randomly distributed using a lottery, and then they were divided into five groups: Group I: root canals preparation and fillings with AHP sealer; Group II: root canals preparation and fillings with EndoSequence BC sealer; Group III: root canals preparation and fillings with BC HiFlow sealer; Group IV (Negative Control): without root canals preparation; and Group V (Positive Control): canals preparation without root canals filling.

Inclusion criteria were mandibular premolars free of caries, single canal, single root, free of cracks and fractures, straight, no open apex, and had not undergone previous endodontic treatment. While exclusion criteria were evidence of internal or external absorption and the size of the apex greater than #20. A radiograph was performed in the buccal-lingual direction to ensure that the canal system was free of any abnormalities and that it was free of internal absorption.

The sample was collected from the department of oral and maxillofacial surgery and immersed in 10% formalin for 24 minutes and then immersed in saline until the start of the study. The crowns were cut with diamond discs, to standard the length of 16 mm and a working length of 1 mm less, then a glide path was established by #10 K-file (Dentsply Maillefer). Root canals were prepared using rotary ProTaper Universal files (up to F2 file) (PTU; Dentsply Maillefer, Ballaigues, Switzerland) by an electric motor (VDW, Munich, Germany) at 300 rpm and 2.5 Ncm. Irrigation with 5.25% sodium hypochlorite was used during the canal preparation, then ethylenediaminetetraacetic acid (EDTA) 17% was applied for a minute to remove the smear layer, then the final irrigation was done with saline. Then, radiographs were taken again after inserting the gutta-percha master point F2 (Maillefer, Ballaigues, Switzerland) into all the root canals to make sure that the completion of canal shaping.

In EndoSequence BC sealer and BC HiFlow sealer (Brasseler USA) groups, a special head to inject the material into the root canal was inserted until 11 mm of the working length, a little of material was placed on the gutta-percha master point F2 before it was inserted into the canal and the canal was filled with a single cone obturation technique.

 In AHP (Dentsply DE Trey, Konstanz, Germany) group, two equal quantities of the two pastes were placed and mixed until a homogeneous consistency was reached. Then the material was inserted into the canal using Lentulo spiral 3-4 mm away from the apical foramen, a little material was placed on the gutta-percha master point F2 before it was inserted into the canal and the canal was filled with a single cone obturation technique.

**Figure 1:**
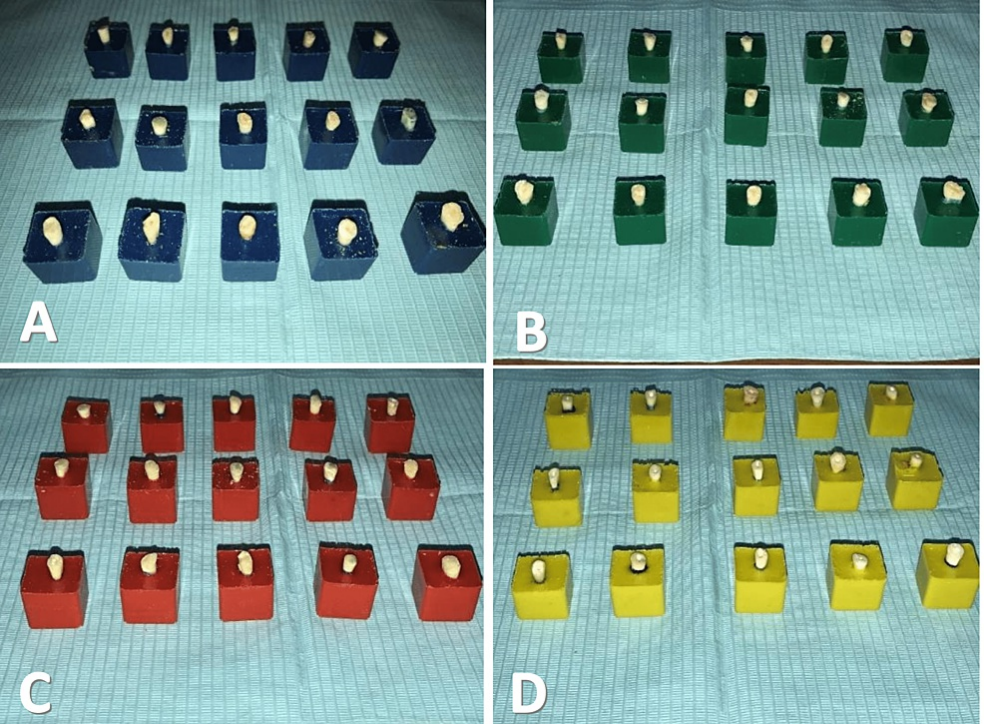
Teeth within the acrylic cube. (A) AH Plus group, (B) EndoSequence Bioceramic group, (C) Bioceramic HiFlow group, (D) Positive Control group

To permit the sealers to be fully set, the samples were placed in the incubator (Asian Universal Testing Machine) for seven days at 37°C and 100% relative humidity, then the roots were covered with a silicone-based material (Zhermack SpA, Italy) to simulate periodontal ligaments, and then each tooth was placed within an acrylic cube (10 mm of each tooth remained visible outside) of dimensions (10 x 10 x 10 mm) without knowing the teeth which group they belong to (Figure 1).

**Figure 2:**
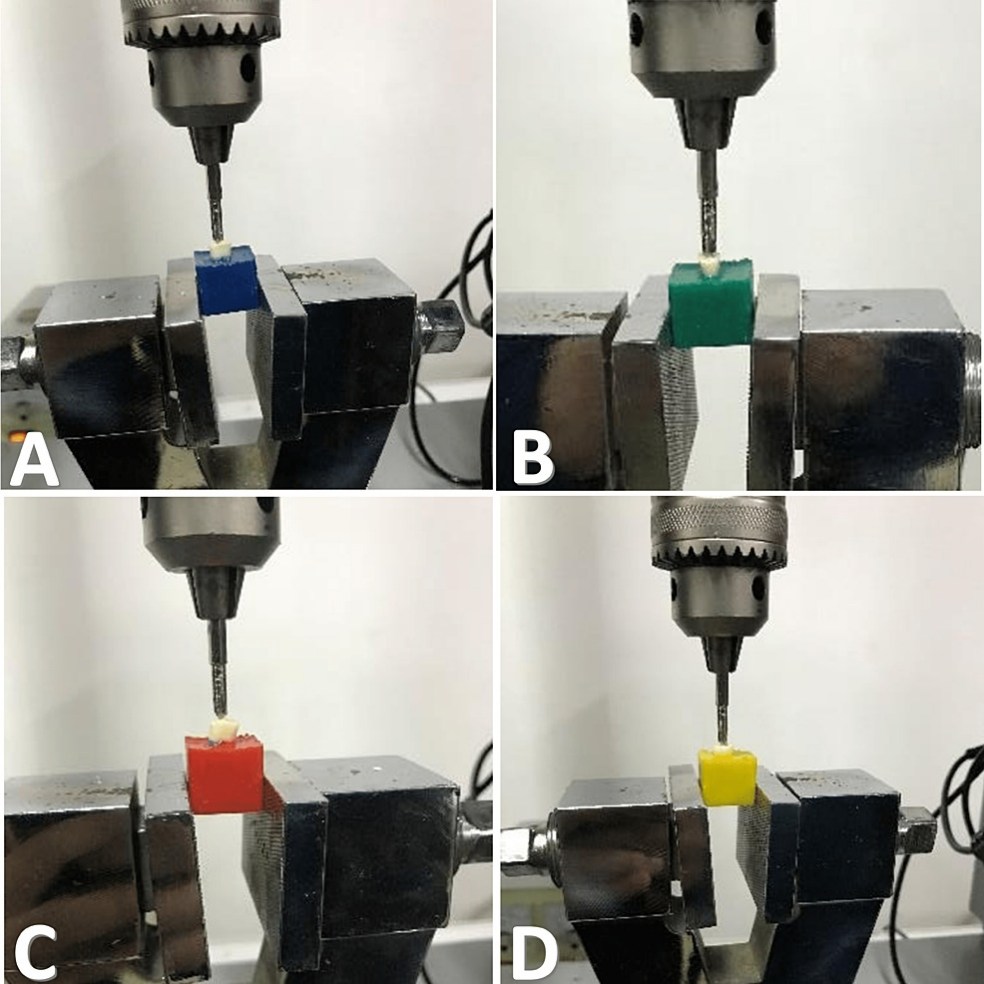
Applying vertical forces parallel to the longitudinal axis of the root using the General Mechanical Testing Device. (A) AH Plus group, (B) EndoSequence Bioceramic group, (C) Bioceramic HiFlow group, (D) Positive Control group

The sample was left for a week to ensure the complete setting of the filling material used, then the acrylic mold holding the root was placed on the base designated for the General Mechanical Testing Device (Testometric, 50Kn, Co Ltd, United Kingdom). Then, vertical forces parallel to the longitudinal axis of the root were applied to the upper surface of the root using a conical tip with a diameter of 2.2 mm and a speed of 1 mm/s until the occurrence of a longitudinal fracture of the root (Figure 2), where the value of the force was recorded at the moment of the fracture. The values were recorded in tables to determine the filler paste with the greatest effect in increasing the root fracture resistance and conducting statistical analysis.

Statistical analysis was performed by using Statistical Product and Service Solutions (SPSS) (IBM SPSS Statistics for Windows, Version 21.0, Armonk, NY). The data were analyzed with the one-way analysis of variance (ANOVA) test. The testing was performed at a significance level of 0.05.

## Results

The study sample consisted of 75 extracted premolars. In order to study the differences in the increase of root fracture resistance between the five groups of the current study sample, the one-way ANOVA test was applied, as shown in Table 1.

**Table 1:** Root fracture resistance values in the studied groups and one-way ANOVA test results (*Statistically differences). BC: Bioceramic; ANOVA: analysis of variance

Studied groups	Number of teeth	Mean	Standard deviation	Min	Max	F-test	P-value
Positive control	15	736.040	99.681	588.2	890.1	49.784	<0.001^*^
Negative control	15	318.040	60.822	185.2	420.7		
AH Plus	15	492.680	70.205	395.3	647.5		
Endosequence BC	15	494.440	86.495	368.4	648.8		
Endosequence BC HiFlow	15	496.960	85.636	374.1	657.8		

Table 1 shows the P-value (<0.001), and therefore there were statistically significant differences in the average measurements of the root fracture resistance between the five studied groups. For pairwise comparisons of root fracture resistance, the Bonferroni test was performed as shown in Table 2.

**Table 2:** Bonferroni test results (*Statistically differences). BC: Bioceramic

Group (1)	Group (2)	Difference between the two means (1-2)	P-value
Positive control	Negative control	418	<0.001^*^
	AH Plus	243.36	<0.001^*^
	Endosequence BC	241.60	<0.001​​​​​​​^*^
	Endosequence BC HiFlow	239.08	<0.001​​​​​​​^*^
Negative control	AH Plus	-174.64	<0.001​​​​​​​^*^
	Endosequence BC	-176.40	<0.001​​​​​​​^*^
	Endosequence BC HiFlow	-178.92	<0.001​​​​​​​^*^
AH Plus	Endosequence BC	-1.76	0.953
	Endosequence BC HiFlow	-4.28	0.886
Endosequence BC	Endosequence BC HiFlow	-2.52	0.933

Table 2 shows a statistically significant difference in root fracture resistance between the positive control group and the other four studied groups (Negative control, AHP, Endosequence BC, Endosequence BC HiFlow), and a statistically significant difference between the negative control group and the three groups (AHP, Endosequence BC, Endosequence BC HiFlow). There were no statistically significant differences in the pairwise comparisons between the three filler pastes (AHP, Endosequence BC, Endosequence BC HiFlow).

## Discussion

Canal preparation is an important stage as it represents an essential stage in endodontic treatment. Endodontic treatment requires a balanced preparation of the root canal system for an impermeable canal filling that ensures long-term success [9]. Several root-filling materials have been suggested, but gutta-percha with pastes is the most widely used as it is considered the most common material because it is characterized by its plasticity, radiopacity, and ease of manipulation [10].

A root fracture is a major clinical problem and when it occurs, not much can be done to save the tooth, and the extraction of the tooth may be the only choice [11]. The main reason for the occurrence of this complication has not been accurately determined, as several hypotheses have been put forward, such as the procedures for preparing and filling the root canal, the application of root cores, and the forces applied when filling with the lateral or vertical condensation technique [12].

Studies have linked root canal preparation with root fracture [13], and it has been proven that all preparation techniques cause root weakening [14]. The filler pastes were previously based on zinc oxide and eugenol, or epoxy resin or glass ionomer cement. Recently, filler pastes with a calcium silicate basis have appeared, such as Endosequence BC and Endosequence BC HiFlow [15]. These pastes are recent and there is still little study available on them. Therefore, this study aimed to evaluate the effect of three filler pastes (AHP, Endosequence BC, Endosequence BC HiFlow), with a single cone obturation technique in the resistance of the endodontically roots fracture.

Calcium silicate sealers produce calcium hydroxide by hydration, which affects water sorption and solubility more than is the case for conventional resin-based sealers, calcium silicate also produces a tag-like structure at the calcium silicate/dentin interface [16].

The mandibular premolars were chosen because of their high incidence of root fractures. The single cone obturation technique was chosen because it reduces the removal of excessive dentin, and the effect of manual reamers using in the lateral condensation technique, and pluggers in warm condensation. Therefore, the forces applied during canal filling were neutralized and the effect of only the filling pastes was studied [17].

The crown was separated from the root so that 16 mm of the root length remained, and a working length of 1 mm less was determined based on the recommendations of Adorno et al., who found that preparation of a working length of 1 mm less than the apical foramen causes fewer cracks on the surface of the apical part of the root [18].

To simulate occlusal forces, a vertical force was applied by a head inserted into the coronal canal orifice recommended by several studies such as Lertchirakarn et al., Wilcox et al., and Lindauer et al. [19-21], this method generates force starting from the inner walls of the root canal. The force-producing head was set to 1 mm/s which is consistent with Sedgley and Messer's study [22], the force at the moment of fracture is recorded in newtons.

The results of this study have shown no statistically significant differences between the three filler pastes (AHP, Endosequence BC, Endosequence BC HiFlow). There were no previous studies that evaluated the effect of Endosequence BC HiFlow on root fracture resistance, but the results of this study agreed with Gervini et al., Varghese et al., and Yendrembam et al., where the resistance of roots to fracture was studied with the use of EndoSequence BC and AHP filler pastes. Studies concluded with results similar to this study, where there were no statistically significant differences [23-25].

The results of this study differed from the study of Patil et al., where EndoSequence BC paste outperformed AHP, with a statistically significant difference. The reason may be due to the difference in the teeth included in the studied sample, where the lower incisors were used, and the Protaper next preparation system was used [26].

The fracture resistance was evaluated by applying a single vertical load parallel to the long axis, and this is the major limitation of the study because in vivo conditions the masticatory forces can be more complicated, and load and forces were in different directions.

## Conclusions

Within the limitation of this in vitro study, it may be concluded that (EndoSequence BC - BC HiFlow- AHP) sealers were able to increase the force to fracture against vertical forces in single-rooted endodontically treated premolar teeth when compared to the prepared roots without canal fillings. While the application of EndoSequence BC, BC HiFlow, and AHP did not increase the fracture resistance of roots compared to that of unprepared root canals.
